# Clinician performed resuscitative ultrasonography for the initial evaluation and resuscitation of trauma

**DOI:** 10.1186/1757-7241-17-34

**Published:** 2009-08-06

**Authors:** Lawrence M Gillman, Chad G Ball, Nova Panebianco, Azzam Al-Kadi, Andrew W Kirkpatrick

**Affiliations:** 1 Regional Trauma Services, Calgary Heath Region and Foothills Medical Centre, Calgary, Alberta, Canada; 2 Department of Surgery, Calgary Heath Region and Foothills Medical Centre, Calgary, Alberta, Canada; 3 Department of Critical Care Medicine, Calgary Heath Region and Foothills Medical Centre, Calgary, Alberta, Canada; 4 Department of Emergency Medicine, University of Pennsylvania, Philadelphia, Pennsylvania, USA

## Abstract

**Background:**

Traumatic injury is a leading cause of morbidity and mortality in developed countries worldwide. Recent studies suggest that many deaths are preventable if injuries are recognized and treated in an expeditious manner – the so called 'golden hour' of trauma. Ultrasound revolutionized the care of the trauma patient with the introduction of the FAST (Focused Assessment with Sonography for Trauma) examination; a rapid assessment of the hemodynamically unstable patient to identify the presence of peritoneal and/or pericardial fluid. Since that time the use of ultrasound has expanded to include a rapid assessment of almost every facet of the trauma patient. As a result, ultrasound is not only viewed as a diagnostic test, but actually as an extension of the physical exam.

**Methods:**

A review of the medical literature was performed and articles pertaining to ultrasound-assisted assessment of the trauma patient were obtained. The literature selected was based on the preference and clinical expertise of authors.

**Discussion:**

In this review we explore the benefits and pitfalls of applying resuscitative ultrasound to every aspect of the initial assessment of the critically injured trauma patient.

## Introduction

Traumatic injury continues to be a worldwide burden on all societies. It is the leading cause of death from ages 15 – 44 in Canada[1], and an ever increasing cause of death and disability in the developing world. In fact, traumatic injury may soon outpace infectious diseases as a leading cause of worldwide mortality[2]. It currently constitutes 16% of the world's burden of disease and is projected to climb as the world continues to industrialize[2]. Road traffic accidents will represent the third leading cause of morbidity and mortality in an "optimistic" baseline prediction of health in 2030[3]. These deaths remain potentially preventable and thus demand urgent attention. Timeliness is critical in trauma resuscitation as many injuries may be completely survivable when addressed quickly yet confer death when not. The remarkable progress in the field of imaging has revolutionized the approach to the critically injured patient, however these technologies continue to be fraught with limitations. While many powerful imaging technologies may be available in a referral hospital, these are typically not located at the resuscitative bedside and are therefore limited only to the hemodynamically stable patient. In addition, they are often absent in smaller referral centres, as well as in the pre-hospital environment. Further, in the settings where the majority of the world's trauma victims are treated, these adjuncts simply do not exist. With the continued advances in ultrasound technique and technology, this may represent an ideal solution to many of these challenges.

Developments in technology make ultrasound a rare example of a medical tool that is rapidly becoming less costly, easier to use, more powerful, as well as portable to the point of being truly hand-carried. In the early minutes of trauma care, ultrasound can assist the clinician by combining the physical examination with a focused goal-directed test that can immediately confirm or refute life-threatening diagnoses. We thus believe clinicians should embrace clinician performed resuscitative ultrasound (RUS) to guide almost every aspect of bedside trauma resuscitation to improve patient care[4,5]. Although RUS is typically interpreted in real-time analog format, it also represents anatomy and physiology captured in a digital format which can be easily archived[4,6].

## Methods

A review of the medical literature was performed using Pubmed and articles pertaining to ultrasound-assisted assessment of the trauma patient were obtained. The references of these articles were reviewed in order to locate additional articles. The literature selected was based on the preference and clinical expertise of authors. The review specifically focused on the early resuscitation and assessment of the injured patient, recognizing an almost unlimited scope of US to assess injured patients throughout their hospitalization.

## Discussion

### Incorporating US as an additional physical exam dimension

The scope of ultrasound in medicine is virtually unlimited. Currently, almost every specialty recognizes both general and unique applications, as well as their ability to increase patient safety[4,7]. Similarly, RUS complements nearly all aspects and diagnostic questions that must be rapidly addressed during the primary ATLS survey[8]. We, like many, consider the RUS to be an extension of our comprehensive physical examination, and attempt to map it to our resuscitative sequence modeled upon the American College of Surgeons' Advanced Trauma Life Support (ATLS) recommendations. True pulseless electrical activity (PEA) can be easily distinguished from cases with residual electrical activity[9,10]. Observing the beating heart in such a situation is simply the visual more powerful equivalent of hearing heart sounds. Similarly, viewing the pleural movement is akin to hearing breathe sounds[11]; seeing multiple pulmonary comet-tails artifacts is akin to hearing lung crepitations[12]; and viewing fluid between previously normal intra-peritoneal organs often confers the benefits of having sharply aspirated the peritoneal cavity[13,14]. While CT scanning or MRI imaging bestow much more diagnostic information with higher image fidelity, these modalities remain "tests" to be ordered by a clinician and constitute remote dangerous locations to which a patient must be reluctantly transported. US is a safer modality that allows one to see deeper than the skin and infer simple yet critical physiology at the bedside, where it can be interwoven with the concurrent assessment and resuscitation required by the ATLS philosophy.

We believe US should be taught as part of the basic skill-set of the physician [15-17], certainly as part of the physical examination, and ideally incorporated as part of anatomy to bring the internal viscera to life[15,16,18]. Most non-surgical physicians will *never *hold a kidney in hand during their careers, but we believe most *will *image the kidney during an office blood-pressure assessment. The challenge for trauma however, is to remain focused, using RUS to enhance decision-making and not to prolong the resuscitation sequence with needless delay. The goal is to detect and manage life-threatening pathophysiology without completing a comprehensive US catalogue[5].

When a question such as the presence of apnea, tension pneumothorax, or hemoperitoneum is obviously and conclusively answered, further detail may in fact be unwarranted. Herein we attempt to describe major attributes of resuscitative focused US, recognizing that there has been an explosion of newly described techniques and experience, and that every clinician will, with practice, develop their own methodology and preferences. We describe the FAST exam as a starting point for discussion, although its use RUS would logically map to an A, B, C, D, E ATLS resuscitation sequence. Further, although RUS may be invaluable in numerous areas of the detailed secondary survey by diagnosing appendicular and axial fractures[19,20], aiding in reduction[21], guiding vascular access[22], this report will focus only on the use of RUS to address the primary survey of trauma.

### The FAST Exam as the basic building block

The Focused Assessment with Sonography for Trauma (FAST)[23] is the most studied example of focused clinical US in trauma care[13,24-28]. FAST has been defined by consensus conference to designate an expeditious, focused interrogation of the pericardial and peritoneal space looking for free fluid as a marker of injury[23]. This exam has gained wide acceptance as a necessary emergency room skill and has been incorporated into the ATLS course for Doctors [29]. Practice management guidelines from the Eastern Association for the Surgery of Trauma recommend FAST as the initial diagnostic modality to exclude hemoperitoneum[30].

**Figure 1:**
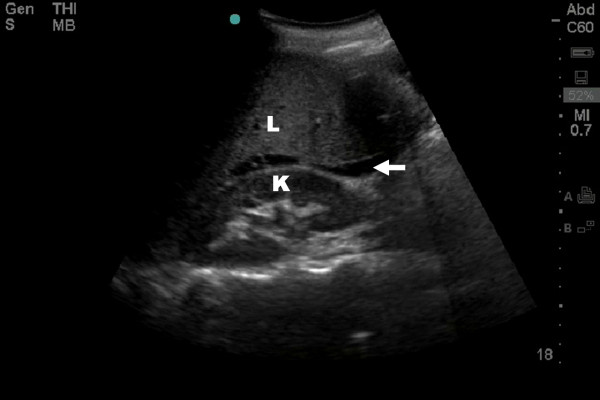
**Resuscitative ultrasound image of hepatorenal fossae demonstrating free intra-peritoneal fluid seen as a hypoechoic stripe (arrow) between the liver (L) and kidney (K)**.

This importance derives from the ability of US to non-invasively "see" the presence of major intra-peritoneal fluid in those with hemorrhagic shock as a result of intra-peritoneal injuries. FAST has been shown to be a highly effective tool in the unstable patient with massive hemoperitoneum as the cause of hemodynamic instability[13,31,32]. In the unstable patient, a massive hemoperitoneum can be quickly detected with a single view of the hepatorenal space (Morison's pouch) in 82–90% of patients[31,33], requiring only 19 seconds on average[31] (Figure 1, Additional file 1: FAST Movie.avi). In order to be comprehensive, and to make a negative determination, the FAST interrogates a minimum of three gravitationally dependant intra-peritoneal windows; the hepatorenal space (Morison's pouch), the splenorenal space, and the pelvis or Pouch of Douglas. Bleeding to death has been considered the single most important cause of potentially preventable trauma death [34,35] with intra-abdominal injury, including pelvic injury, representing the most common single site of inaccessible exsanguination[35,36]. This infers that preventing shock by arresting hemorrhage remains the most important single task of any physician committed to provide emergency medical care[5]. The fact that intraperitoneal fluid can be demonstrated in seconds allows expeditious operative planning and obviates the need for further testing in the unstable patient. Randomized trial has demonstrated that FAST reduces time to operative care in those requiring surgery[37]. Alternatively, if the FAST is truly negative in the hands of a competent user, and not just indeterminate[38], this directs the search for the source of hemorrhage towards the retroperitoneal cavity where unstable patients may be better cared for in an angiography suite rather than the operating room[39].

**Figure 2:**
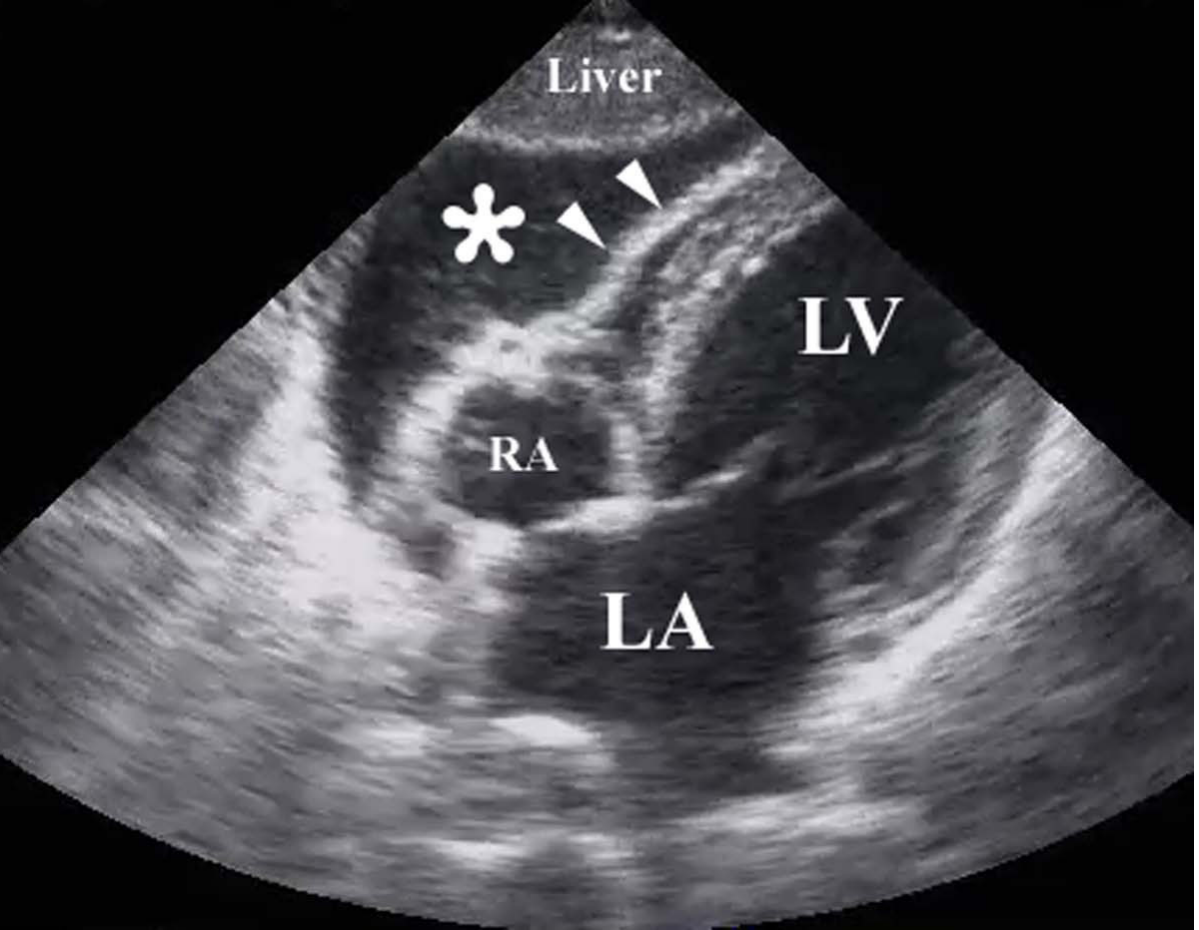
Resuscitative ultrasound image following a penetrating chest injury illustrating the presence of a pericardial tamponade from a hemopericardium (*). Arrowheads illustrate the wall of the right ventricle. RA – right atrium, LA – left atrium, LV – left ventricle.

An interrogation of the pericardium represents the fourth component of a complete FAST examination (Figure 2)[13,27,32]. This portion of the examination is especially useful and potentially life-saving in penetrating injuries[40], although negative examinations are considered non-helpful[41].

We thus believe that any physician caring for the trauma patient should be competent in the FAST examination. Similar opinions have supported many educational endeavours and programs for adult learners first encountering ultrasound, making the FAST exam a common "starting point" for bringing US into an established practice. We do not use the FAST as a standalone test in the hemodynamically stable[26,28,42], considering it to be part of our physical examination and thus complementary to other imaging modalities especially CT scan.

### Intra-pleural Fluid – Hemothoraces

**Figure 3:**
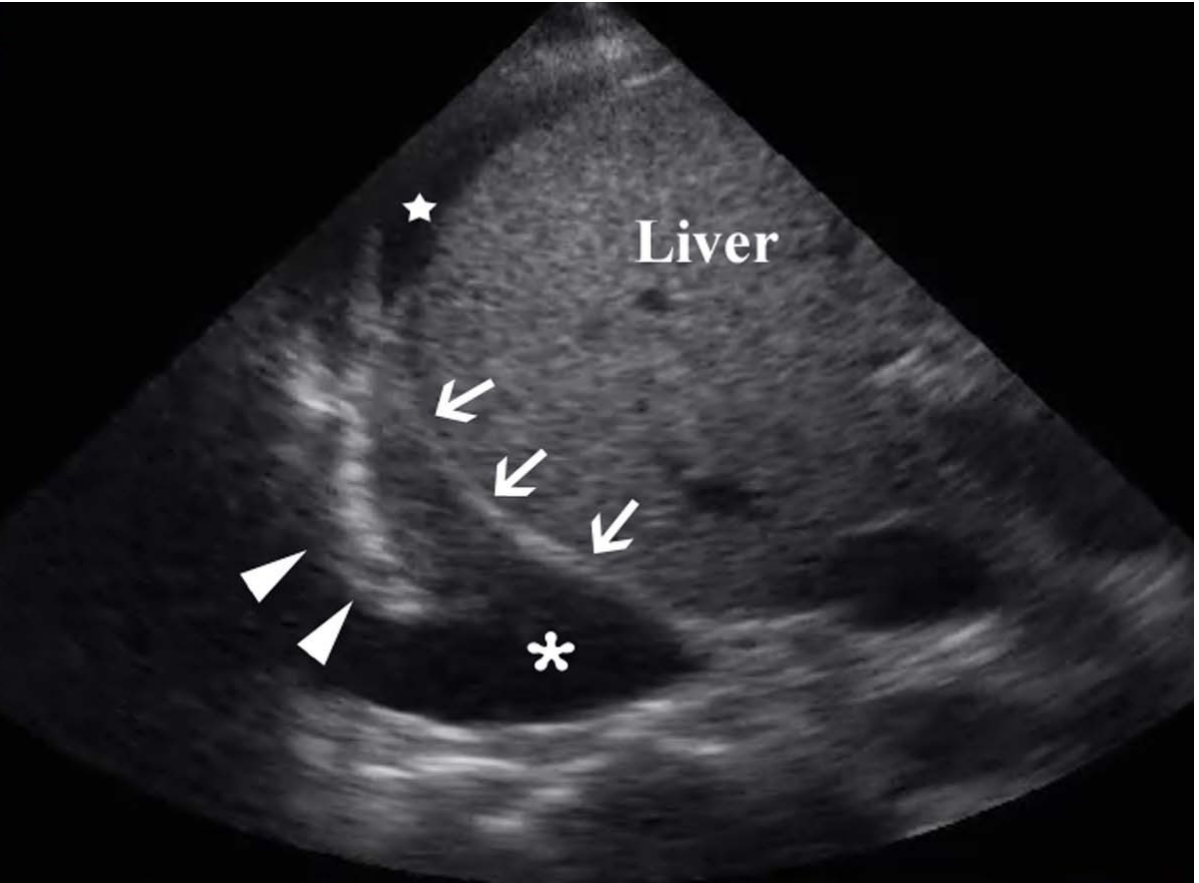
Resuscitative ultrasound image of large pleural collection (*) seen here above the diaphragm (arrows) around the collapsed right lower lobe (arrowheads). Note also the free intra-abdominal fluid (Black star).

**Figure 4:**
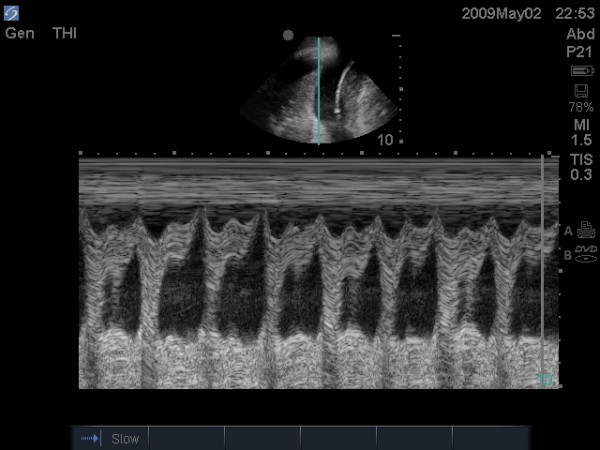
**Resuscitative ultrasound image using time motion mode demonstrating the sinusoid sign**. This sign illustrates an undulation of the collapsed lung tissue within the pleural fluid thus confirming the fluid nature of the intra-pleural contents.

Once clinicians are comfortable imaging the upper abdomen, it is natural to add an assessment of the lung bases and diaphragms for intra-pleural fluid (Figure 3). This can easily be done without switching from the abdominal probe in hand. Sisley and colleagues[43] demonstrated that when using the same probe, US was 97.5% sensitive and 99.7% specific compared to chest radiograph's 92.5% and 99.7% respectively. Similarly, Ma also demonstrated a 96% sensitivity and 100% specificity[27]. This experience has led many investigators to augment the standard FAST exam with routine views of the pleural space. The presence of pleural fluid such as a massive hemothorax can be quickly confirmed and documented by either simple pattern recognition (Figure 3, Additional file 2: pleural fluid.avi), or by capturing the respiratory variation in the contours of the intra-pleural fluid known as the sinusoid sign when M-mode (time motion mode) is used (Figure 4)[44]. This mode, standard on most basic machines, displays one line of the ultrasound window as it changes over time. In this case, it demonstrates the respiratory oscillation of the collapsed lung within the sea of hemothorax.

### The Extended FAST (EFAST) for Inferred Pneumothoraces

The term EFAST or extended FAST was coined by our group in 2004 to denote not only a rapid assessment of the abdomen and pericardium for free fluid but also an assessment of the chest to rapidly rule out a pneumothorax[45]. This very principle underscored the definition of FAST as denoting focused assessment with sonography for trauma rather than focused abdominal sonography for trauma[23]. Although currently the terminology denotes the FAST as the "usual" abdominal exam and EFAST as adding a search for pneumothoraces, we anticipate a future where this distinction blurs, and a comprehensive yet focused resuscitative exam is simply performed appropriate for the setting and injuries[8].

Ultrasound of the lung fields is a relatively new modality. For years it was thought that ultrasound of the lung was impossible given that ultrasound beams are highly attenuated in air. In fact, Harrison's textbook of medicine concluded that "ultrasound imaging is not useful for evaluation of the pulmonary parenchyma" [46]. Over the past two decades however, it has been realized that while ultrasound beams may be reflected by an air-tissue interface, an interpretation of the artifacts that this reflection creates can yield a great understanding of the status of the underlying pleural space and lung parenchyma. Further, much of the immediate life-threatening pathology that needs to be detected early in resuscitation is pleural based and thus accessible to RUS such as massive hemothoraces (as demonstrated earlier) and tension pnumothoraces[47]. This new role for ultrasound in imaging of the lung has led to an explosion of literature on the topic over the past few years.

**Figure 5:**
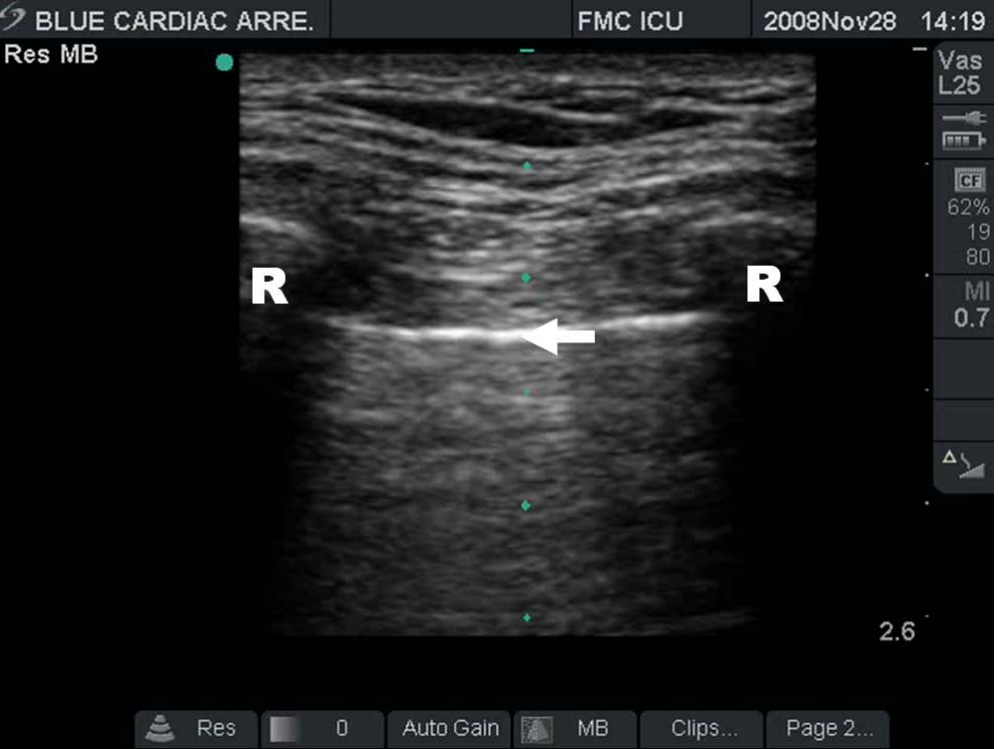
**Resuscitative ultrasound image illustrating the batwing sign**. The pleural line is seen approximately 0.5 cm below the rib shadows on either side.

In the supine trauma patient, the intra-pleural air of a pneumothorax is typically found anteriorly within the pleural space[48]. Therefore, the site to first examine for a pneumothorax, is the same site where a pneumothorax would be needle decompressed, at approximately the 2^nd ^interspace in the mid-clavicular line. The probe is placed longitudinally on the chest wall spanning the second interspace perpendicular to the direction of the ribs. The ribs are identified by their characteristic posterior shadowing, and 0.5 cm below two contiguous ribs, a hyperechoic line can be seen. This line, denoted as the "pleural line", represents the parietal and visceral pleural interface. Together, the upper rib, pleural line, and lower rib form a characteristic pattern, the bat wing sign[49] (Figure 5). Identification of this sign prevents confusion with subcutaneous emphysema which will appear similar to air in the pleural space but will lack the characteristic rib shadowing above.

In a healthy patient one can visualize a to-and-fro movement at the pleural line that is synchronized with respiration (additional file 3: lung sliding.avi). This to-and-fro movement is called lung sliding[44,45,49-52] and is caused by the movement of the mobile visceral pleural along the static parietal pleura. We consider this depiction as the visual equivalent of hearing breath sounds[14]. If air is interposed between the pleura the ultrasound beam would be reflected by this air and would not penetrate to the underlying visceral pleura and therefore normal lung sliding would be absent (additional file 4: absent lung sliding.mov). Hence the presence of lung sliding implies apposition of the visceral and parietal pleura by definition ruling out a pneumothorax[51].

**Figure 6:**
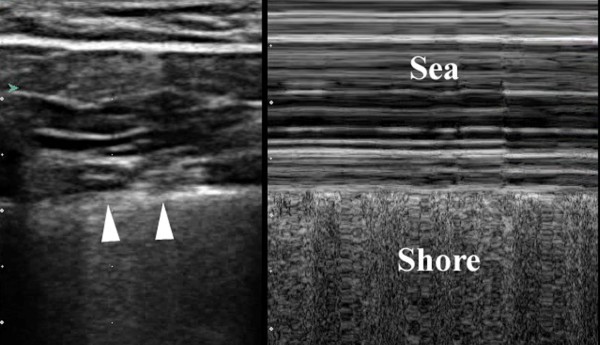
**Split field image demonstrating static 2-D mode depiction of normal pleura (arrowhead) on the left of the image, with M-mode depiction of the sea-shore sign on the right side of the image**.

Often lung sliding can be subtle, especially anteriorly in the upper chest. Therefore other methods have been described to emphasize the depiction of sliding. One such method utilizes the M-mode of the ultrasound machine[49,53,54]. In the presence of sliding, use of the M-mode reveals parallel lines above the 'pleural line' that correspond to the motionless parietal tissue of the chest wall. Meanwhile below the 'pleural line' a homogenous granular pattern is seen corresponding to the constant motion of the underlying lung and giving the appearance of a sandy beach intersecting with rolling waves in a pattern known as the 'seashore sign'. This confirms the presence of lung sliding (Figure 6) [49,53,54].

**Figure 7:**
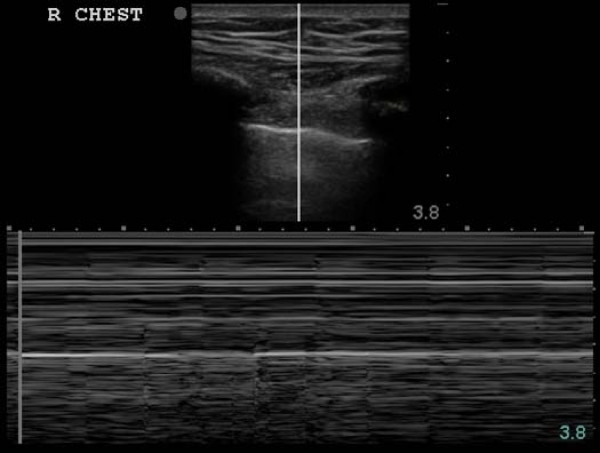
**Stratosphere sign of a pneumothorax; 2-D image above indicates line of interrogation of pleural interface**. The corresponding time motion mode image fails to reveal any underlying pleural movement consistent with a pneumothorax.

**Figure 8:**
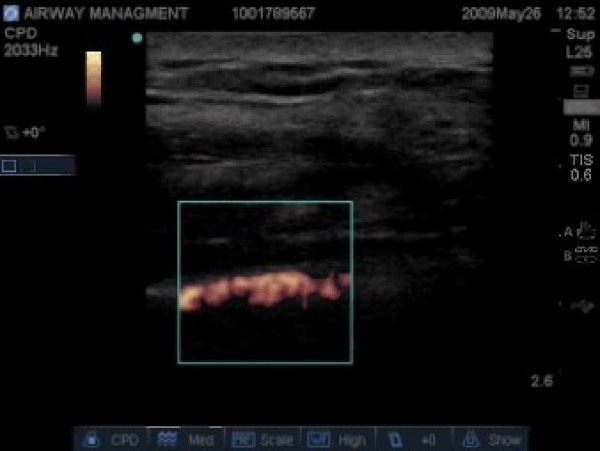
**Color power Doppler image illustrating the presence of movement at the pleural line – thus confirming lung sliding**.

In the case of a pneumothorax, this normal sliding is absent and M-mode reveals a series of parallel horizontal lines, suggesting complete lack of movement both over and under the pleural line. This is known as the 'stratosphere sign' (Figure 7). [49,53,54]. A second method used to enhance the recognition of lung sliding utilizes color power Doppler (CPD). CPD is superior to regular color Doppler in determining the presence or absence of movement at the expenses of directionality and velocity[55]. Color enhancement of the pleural line sliding with respiration is known as the 'power slide' (Figure 8, Additional file 5 – power slide.avi) [6,54].

A number of studies have looked at the sensitivity and specificity of lung ultrasound for the diagnosis of pneumothorax. Using the absence of lung sliding as a definition for pneumothorax the sensitivities range from 80 to 98% and specificities from 91 to 99%[49,51,56-60]. One of the pitfalls of this technique has been bilateral pneumothoracies, likely due to the loss of a patient-specific "normal" comparative examination[45]. In addition, as mentioned earlier, diffuse subcutaneous emphysema can make visualization of the underlying pleural line impossible, also interfering with ultrasound diagnostic ability. This potential pitfall is relatively unimportant in the unstable patient, as subcutaneous emphysema equates to an underlying post-traumatic pneumothorax in our experience[61].

**Figure 9:**
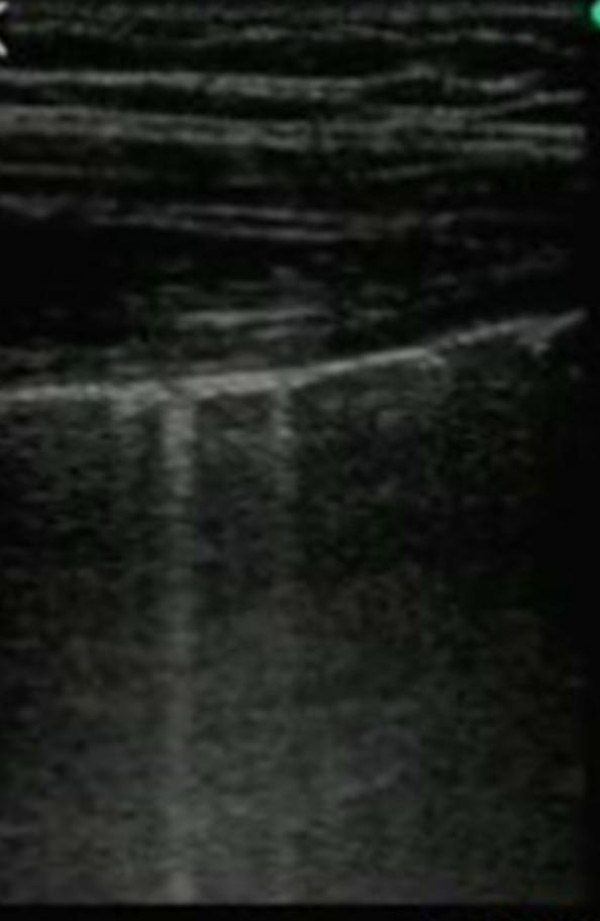
**Resuscitative ultrasound image illustrating comet tail artifacts**.

Comet tail artifacts, B-lines or lung rockets are designators of US artifacts created by the presence of thickened interlobular septa in the underlying lung parenchyma (Figure 9, Additional file 6 – comet tail artifacts.avi) [62,63]. While in the normal lung a few scant comet tail artifacts can be seen, these artifacts are generally more pronounced in cases where these interlobular septa are thickened by edema including acute respiratory distress syndrome, pulmonary edema, pulmonary contusion, and aspiration [64-66]. Since these artifacts originate from the parenchymal surface of the lung, their presence within the ultrasound field implies the apposition of the parietal and visceral pleura, and also rules out a pneumothorax with a negative predictive value approaching 100%[58,62]

**Figure 10:**
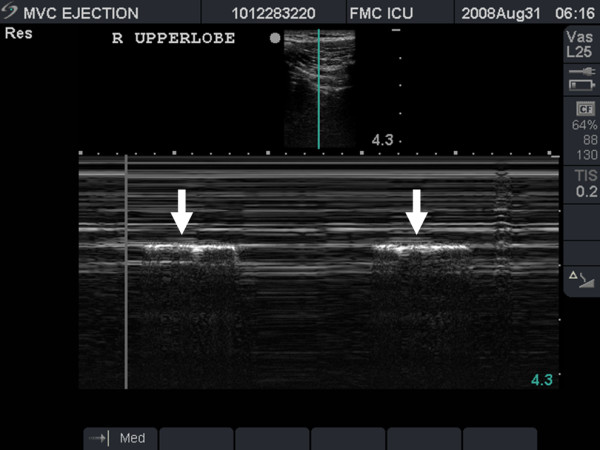
**Resuscitative ultrasound image illustrating a lung point in time motion mode**. The normal seashore sign (arrows) can be seen alternating with the stratosphere sign in time with respiration.

While ultrasound's main strength lies in its ability to rule out the presence of a pneumothorax, in certain situations it also can be used to confirm the presence a pneumothorax. Once a pneumothorax is suspected, the probe should be scanned laterally along the chest wall in an attempt to discern the size of the pneumothorax[53,57]. This technique attempts to identify the lateral extent of the pneumothorax by localizing the point on the chest wall where the normal lung pattern can be seen alternating with the pneumothorax pattern (absent lung sliding) in time with respiration. This point is called the 'lung point'. At this position, during expiration, no sliding is seen, but with inspiration, the lung inflates and the visceral pleura moves up in apposition with the parietal pleura beneath the ultrasound probe and sliding is again seen (Additional file 7 – lung point.avi). The ability to demonstrate the alternating lung sliding and absence of lung sliding within the same ultrasound field is diagnostic of a pneumothorax with a sensitivity of 66% and specificity of 100%[53]. This can also be demonstrated using M-mode where one sees alternating patterns of the 'seashore' and 'stratosphere' signs (Figure 10)[53,54].

### Airway Management

Establishment and protection of a patent airway is typically the highest priority in emergency trauma care. The most definitive airway protective maneuver is placement of an endotracheal tube (ETT). Unfortunately, rates of tube malposition double in the emergency situation[67], and success rates for first time intubations in the field by basic emergency medical technicians was found to be only 51%, with a 25% recognized and three percent unrecognized esophageal intubation rate[68,69]. ETT placement can be confirmed by a number of different modalities, including direct observation at laryngoscopy, auscultation of bilateral equal air entry, observation of chest wall excursion, confirmation with capnography, and ensuring adequate oxygenation (although hypoxemia is a late and ominous finding of tube malposition). Capnography has been considered the gold standard but it relies on an adequate cardiac output to deliver CO_2 _and, thus may be inaccurate in a cardiac arrest setting, or simply not available in many pre-hospital settings. Unfortunately even in the best of settings, clinical examination is notoriously inaccurate and receiving physicians cannot be present for pre-hospital intubations yet assume responsibility. Sixty percent (60%) of right mainstem intubations occur with equal breath sounds documented, and 70% occur despite the observation of apparent symmetric chest excursion[70,71].

Fortunately RUS can quickly and easily augment the standard physical assessment of ETT placement. Firstly, the location within the trachea can simply be visualized directly as primary confirmation of ETT placement taking 17 seconds on average versus 14 minutes for a subsequent chest radiograph in one study [72-74]. While expedient, this methodology does not rule out a right main-stem intubation however. The presence of secondary signs of ventilation such as lung sliding, the movement of comet-tail artifacts, and a positive power slide may provide this information though [11,74,75]. These signs can be noted with a simple view of the left lung, and can thus assure one that the left lung is moving. This simple fact simultaneously rules out an esophageal and a right main stem intubation in these patients. Several authors have utilized a similar philosophy by bilaterally imaging the diaphragmatic movements after intubation from a subxiphoid window to rule out a right main-stem intubation[76,77]. None of these techniques verify the adequacy of ventilation though, emphasizing the need to always incorporate RUS findings with the entire clinical picture.

### Focused Examination of Cardiac Function

After airway and breathing issues have been addressed, clinicians next focus on assuring adequate circulation. While the basic FAST limits its evaluation to the pericardial sac, a number of groups have recognized that RUS can potentially add critical information regarding cardiac function and volume status. Formal echocardiography is a complex technical and cognitive skill requiring extensive training and practice. With modest training though, non-expert cardiographers can quickly learn valuable details regarding their patient's physiologic status with simple, focused examinations that alter management [78-81]. A number of different acronyms and protocols have been suggested to designate goal directed cardiovascular examinations by non-dedicated echocardiographers including the focused assessment with transthoracic echocardiography (FATE)[80], bedside limited echocardiography by the emergency physician (BLEEP)[82], bedside echocardiography assessment in trauma/critical care (BEAT)[83], or descriptive summaries emphasizing goal-directed, limited, or focused examinations[78,84]. A number of resuscitative protocols have been recently formalized for the patient with undifferentiated hypotension. These describe combinations of an abdominal assessment for free fluid, qualitative cardiac and inferior vena caval assessment for volume status and cardiac function, and an evaluation of the abdominal aorta for evidence of aneurismal rupture[85,86]. All these approaches are based on the underlying premise that acute sonographic findings are not subtle in the critically unstable patient, and that clinical sonographers recognize their limits with these screening examinations. Dedicated studies are then obtained when time permits or the clinical answers are not obvious. Rose emphasizes ruling in obvious pathology rather than definitively excluding conditions not seen[85]. Complete descriptions of the scope of focused echocardiography are beyond this review, but can be found in a number of other more comprehensive sources[79,80,87,88]. Collectively, these studies support that physicians with basic ultrasound skills who undertake limited but focused training in echocardiography can estimate gross ventricular function with acceptable accuracy when assessed categorically[81,82,84,89,90], and that educating point of care providers in these skills is a future priority.

### IVC Diameter and Volume Status

A focused assessment of the inferior venous cava (IVC) may be expeditiously performed and integrated into the combined ultrasound and clinical picture. While classic trauma teaching suggests that all hypotensive patients are hypovolemic until proven otherwise, not infrequently trauma patients with other non-volume related causes of shock are seen. In addition, the only sign of hemodynamic compromise in a young otherwise healthy trauma patient may be a sinus tachycardia, which can occur with a multitude of other causes including pain, as well as agitation related to substance use or withdrawal. Invasive methods to assess volume status include the placement of central venous lines with measurement of central venous pressure (CVP), or pulmonary artery catheters. These have obvious disadvantages including a delay in placement and potential complications. Sonographic measurement of IVC diameter has been suggested as an alternative, rapid, non-invasive method of volume assessment. This technique has been previously used with good success to estimate volume status in patients undergoing hemodialysis[91,92]. An elegant study by Blaivas et al. revealed a statistically significant 5 mm decrease in IVC diameter was seen after donation of 450cc blood in healthy donors[93].

**Figure 11:**
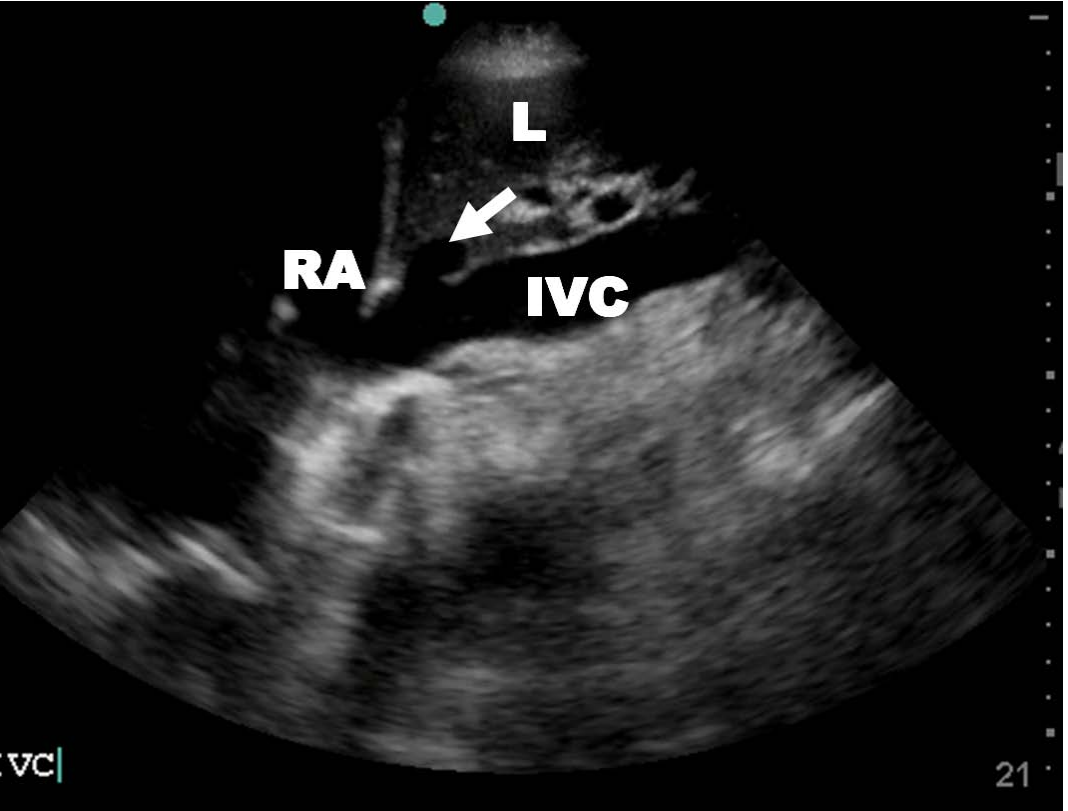
**Resuscitative ultrasound image illustrating a subxiphoid view of the inferior vena cava (IVC) and hepatic vein (arrow)**. RA – right atrium, L – liver.

The IVC is visualized through a subxiphoid or a right lateral window at the midaxillary line depending on patient body habitus and interference of bowel gas (Figure 11). With the patient in the supine position, the diameter of the IVC is measured at both end inspiration and end expiration if breathing spontaneously. In ventilated patients as the relationship between the respiratory cycle and IVC dynamics remains controversial; minimal and maximal IVC diameters may be used[94]. These measurements are taken within 2 cm of its entrance into the right atrium. As with CVP, there are no clear normal values that can be assumed in all patients without exception. Hypovolemia is increasingly likely with IVC diameters smaller than 1 cm however [95-97].

Another parameter that has been assessed as a measure of intravascular volume status is the IVC collapse index (IVC-CI). Under normal conditions in healthy spontaneously breathing patients the IVC will collapse with inspiration and expand with expiration. The IVC-CI is calculated using a standard formula IVC-CI = (IVCDmax) – (IVCDmin)/(IVCDmax) [98], where IVCDmax is the maximum IVC diameter and IVCDmin is the minimum one. The respiratory variation has been found to be more pronounced in hypovolemia with abnormally low CVP being increasingly likely as IVC-CI approaches 100%(Additional file 8 – collapse IVC.avi)[98,99]. Unfortunately there is still no clear consensus on an exact IVC-CI cutoff for hypovolemia.

Inferior vena cava diameter shares many of the same limitations as CVP measurements. These include unpredictable variations with positive pressure ventilation, as well as elevation in right heart failure, valvular disease and pulmonary hypertension unrelated to volume status. Another potential limitation may be abnormal narrowing of the IVC in patients with elevated intraabdominal pressure[100]. One study tried to augment the value of IVC diameter by following it dynamically over time as fluid resuscitation continued in patients who initially presented with shock. This would mimic a similar use of CVP measurements where a single value is sometimes useful but the more helpful situation would be to follow the dynamic change of CVP in response to fluid resuscitation[101]. In this one small study, while all patients initially responded hemodynamically to fluid resuscitation, those with smaller IVC diameters following volume administration were more likely to be transient responders and require surgical intervention[95].

### Focused Ultrasound in Closed Head Injury

**Figure 12:**
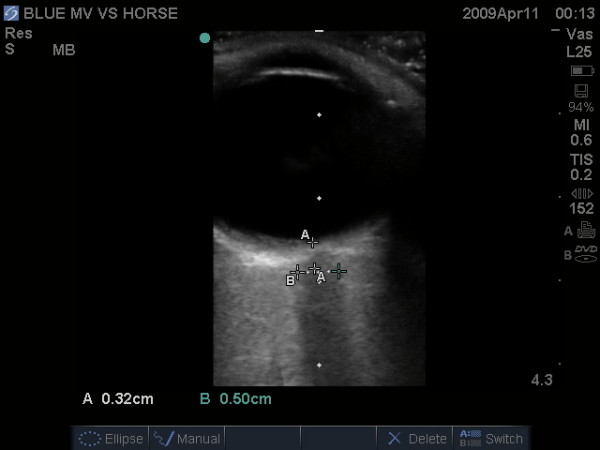
**Resuscitative ultrasound image illustrating measurement of the optic nerve sheath diameter (3 mm behind the globe) in a patient with elevated intracranial pressure**.

Civilian trauma resuscitation in the Western world is confronted with the challenge that head injuries constitute the single largest cause of post-traumatic death[102]. The resuscitation of serious closed head injuries thus demands the early identification of intracranial hemorrhage causing elevated intra-cranial pressure (ICP) amenable to surgical intervention. While ICP monitoring is recommended in patients with decreased Glasgow Coma Scale (<8) and an abnormal CT scan[103], hemodynamic instability from associated injuries requiring operative intervention can result in significant delays in both imaging and monitor insertion. As such, attempts have been made to develop a rapid, noninvasive method of ICP assessment. Even after resuscitation, increased ICP is associated with poor outcomes[104]. It is possible that clinicians can quickly infer raised intra-cranial pressure from an early focused examination of the optic nerve sheath diameter (ONSD), as the optic nerve sheath is anatomically continuous with the dura mater through which cerebrospinal fluid percolates[105,106]. As a result intracranial pressure changes are transmitted through the subarachnoid space to the optic nerve[107]. The actual technique has been relatively standardized[108,109]. Patients are placed in a supine position and a thick layer of gel is applied over the closed upper eyelid. A high frequency linear probe is placed gently on the temporal area of the eyelid and the entry of the optic nerve into the globe is visualized. A reference position 3 mm behind the globe is chosen to give the greatest US contrast, being the most distensible part of the sheath, and giving the most reproducible results (Figure 12) [110,111]. For each optic nerve two measurements are made–one in the sagittal plane and the other in the transverse plane–by rotating the probe ninety degrees.

Most recent series of injured patients have shown good correlation between raised ICP[108], as well as the presence of severe brain injury and neuroimaging result[109,111]. The ONSD has been shown in multiple studies to correlate with intracranial pressure measurements[108,111,112]. While experience with this technique increases, there has yet to be any large studies to determine an appropriate normal range. Normal reference ranges have been considered up to 5.0 mm in adults, 4.5 mm in children aged 1 to 15, and 4.0 mm in infants[105,106], although newer series have suggested that 5.7 mm might be better discriminators of worrisome dilatation[108,111].).). Those studies using the mean ONSD agree that an initial ONSD of less than 5.0 mm correlates with a normal ICP measurement (<20 mmHg)[112]. Meanwhile, a measurement of >5.0 mm has been shown to correlate with elevated ICP (>20 mmHg) [111,112] or evidence of increased ICP on CT scan of the head[109,113]. The value of this technique has yet to be correlated with eventual outcome and recovery. Soldatos found that ONSD did not correlate with estimated ICP by trans-cranial Doppler in either moderate brain injury or controls, and suggests that the optic nerve sheath has a baseline constant diameter that remains constant as long as ICP remains within a normal range[111]. However, optic nerve sheath diameter represents a potentially quick, noninvasive technique to estimate ICP in unstable trauma patients utilizing readily available equipment and requiring a minimal amount of training. It has all the advantages of ultrasound including being easily repeatable for dynamic changes over time. We would finally note that of all the components of the EFAST, we have found this exam to require a significant amount of experience and practice.

### Probe Selection

A multitude of ultrasound probes are now available, each designed with a specific purpose in mind. For our purposes these probe types can be divided into three distinct groups based on the shape of the ultrasound beam produced: (1) linear, (2) curvilinear and (3) phased arrays. In addition, the depth of penetration and resolution of any probe are inversely proportional to each other and determined by the frequency of the ultrasound probe. Higher frequency probes have excellent resolution for near objects but poor overall penetration, while low frequency probes sacrifice some resolution in order to penetrate to deeper structures.

In the classical description of the FAST exam a low frequency curvilinear probe was used [23,25]. This allows for excellent penetration to the deeper abdominal organs but does make imaging of the heart somewhat difficult because of its large footprint. This is also an ideal probe for imaging of the IVC for volume status assessment. Compared to the abdominal probe, the phased array, which is classically used for echocardiography, has a midrange frequency, but a much smaller footprint allowing better visualization of the heart between the ribs. It has been our experience that this probe actually provides reasonable abdominal visualization as well, and may rival that of the low frequency curvilinear probe. Therefore the phased may represent a reasonable alternative probe for the FAST exam allowing for better visualization of both the heart and the intraabdominal structures though to our knowledge this has never been evaluated in the literature.

Although any ultrasound probe will suffice for a rapid assessment to rule out a pneumothorax a high frequency linear probe [45] or a microconvex linear probe [49,50] are the two most commonly used, as they provide the best definition of the pleural interface. The high frequency linear probe is also the only probe described for measurement of the optic nerve sheath diameter [111,112].

For the purpose of a rapid assessment of the multi-injured trauma patient, ideally a single probe could be used for the imaging of all body systems[114]. Unfortunately, in our opinion, this ideal, multi-versatile probe still does not exist. However, we do find that the combination of two commonly available probes is quite sufficient. Either a low frequency curvilinear probe or phased array to assess for free intra-abdominal fluid and hemothorax, and assess the heart, pericardium and the IVC diameter, combined with a high frequency linear probe (commonly used also for line insertion) to rule out a pneumothorax and assess for the presence of intracranial hypertension.

### Truly hand-carried ultrasound (HCUS)

Truly portable HCUS units have become available to clinicians over the last decade with greater industry competition each year. This has resulted in rapid developments in the imaging quality that can be brought to the bedside for RUS. The first such units were developed through a joint civilian-military initiative to provide portable ultrasound capabilities suitable for battlefield or mass casualty situations[115]. These devices allow earlier diagnosis, even in the pre-hospital setting to expedite transport priorities and disposition. This class of ultrasound has been tested in many adverse and austere environments such as military conflicts, land and air ambulance transport, ski-patrols, and during expeditions to the Amazon and Mount Everest. Each has been found to be clinically useful [116-122]. Blaivas and colleagues formally studied the image quality of a first generation HCUS compared to a standard large floor-based machine. They found a statistical difference between the two in resolution and image quality, but not detail as rated by blinded reviewers[123]. It should be noted that they studied a first generation HCUS and that the image quality and overall performance of generations of this and other companies have greatly improved in recent years. Although the fidelity and image-quality of early HCUS units did not match that of standard floor-based machines, their diagnostic performance regarding the FAST examination appears functionally comparable[32,124,125]. With current generations of HCUS, limitations are almost certainly to be related to the operator rather than the equipment.

### Remote clinical guidance

Although emergency medicine programs in particular have widely embraced focused clinical ultrasound, there are innumerable emergency care facilities and settings worldwide in which access to RUS is still lacking. As noted above, as RUS equipment becomes cheaper and more portable every year, it is likely that the trained operator will be the limiting factor. This situation is akin to that on the International Space Station where there is an advanced ultrasound machine, yet no trained operator. This reality prompted the National Aeronautics and Space Administration to study both the extended capabilities of ultrasound to assist in nearly every facet of the physical examination, and the nuances of having remote ground-based experts guide novice users to obtain clinically meaningful images with minimal training [126-129]. We have examined this same philosophy in order to better understand the nature of referred trauma patients between a rural referring centre and a quaternary trauma facility using resuscitating clinicians with a range of RUS experience and noted both clinical and educational benefits[130,131]. Alternate approaches to providing RUS guidance for remote or hostile facilities include remote-controlled robotic tele-US systems[132,133], and three-dimensional ultrasound reconstruction[134,135]. These have currently been developed and trialed in sub-acute or chronic care situations. At present though, robotic systems still require a semi-trained assist at the patient-site, are somewhat unwieldy, and may have difficulty assessing the lateral surfaces of the abdomen including the spleen and kidneys[132,133]. To date there are no published results regarding the benefits of either of these alternate techniques in acute trauma resuscitation and the simple solution remains to properly educate all clinicians in basic clinical US.

## Conclusion

RUS has become a convenient bedside tool that adds an additional "sense" to the physical examination. It has quickly evolved from the original "focused assessment with sonography for trauma" to a truly holistic examination incorporating an evaluation of nearly every aspect of the multi-injured trauma patient. As ultrasound technology continues to evolve, its scope is quickly becoming limited only by the expertise of the operator rather than the capabilities of the bedside unit. It is now essential that all clinicians involved in the care of the trauma patient be well versed in the techniques of RUS, understanding not only their advantages but also their limitations. This will allow RUS to be seamlessly integrated into the resuscitation sequence without adding needless delay.

## Competing interests

AK is vice president of the Canadian Emergency Ultrasound Society; module co-director of the Residents Ultrasound Course of the National Ultrasound Faculty of the American College of Surgeons; and a Committee Member of the World Interactive Network Focused on Critical Ultrasound.

## Authors' contributions

LG, CB, NP, AA and AW all helped with the literature review and in the drafting of the manuscript. LG, NP and AW also helped with collection of images. All authors read and approved the final manuscript.

## Supplementary Material

Additional file 1 **Positive FAST of Hepatorenal Fossa**. Resuscitative ultrasound video of the hepatorenal fossa demonstrating free intra-peritoneal fluid seen as a hypoechoic stripe between the liver and kidney. Click here for file

Additional file 2 **Pleural Fluid**. Resuscitative ultrasound video of a large pleural collection. Click here for file

Additional file 3 **Lung Sliding**. Resuscitative ultrasound video illustrating lung sliding – the to and fro movement of the visceral pleura on the partietal pleura. Click here for file

Additional file 4 **Absent Lung Sliding**. Resuscitative ultrasound video illustrating the absence of lung sliding suggestive of a pneumothorax. Click here for file

Additional file 5 **Power Slide**. Resuscitative ultrasound video illustrating movement at the pleural line on color power Doppler mode – the power slide. Click here for file

Additional file 6 **Comet Tail Artifacts**. Resuscitative ultrasound video illustrating the multiple comet tail artifacts originating from the pleural surface. Click here for file

Additional file 7 **Lung Point**. Resuscitative ultrasound video illustrating the lung point – the lateral limit of the pneumothorax. The lung can be seen sliding in from the right with respiration. Click here for file

Additional file 8 **Ultrasound Assessment of Volume Status**. Resuscitative ultrasound video illustrating complete collapse of the IVC with respiration, suggestive of hypovolemia. Click here for file
